# Complete Genome Sequence of Serratia marcescens Myophage Moabite

**DOI:** 10.1128/MRA.00741-19

**Published:** 2019-07-18

**Authors:** Lyndsey Price, Matthew Rohren, Heather Newkirk, Mei Liu, Jolene Ramsey

**Affiliations:** a Center for Phage Technology, Texas A&M University, College Station, Texas, USA; DOE Joint Genome Institute

## Abstract

Serratia marcescens is a Gram-negative nosocomial pathogen causing various hospital-acquired infections. Here, we describe the complete genome sequence of S. marcescens myophage Moabite. The genome of Moabite is 273,933 bp long, with 337 predicted coding sequences and two tRNA genes, and it shares its highest amino acid identity with Serratia phage 2050HW.

## ANNOUNCEMENT

Serratia marcescens is a Gram-negative nosocomial pathogen often causing hospital-acquired urinary tract, bloodstream, and other infections (1). Treating S. marcescens infections can prove difficult due to its panresistance, including that to metallo-beta-lactamases (2). Due to this wide range of antibiotic resistance, bacteriophage therapy may be a more effective treatment. To that end, the novel myophage Moabite was isolated, and we present its genome sequence here.

Moabite was isolated from a combination of filtered (0.22 μm) and chloroform-sterilized U.S. swine farm samples based on its ability to grow on S. marcescens D1 (catalog no. 8887172; Ward’s Science). Both the host and phage were cultured as described by Adams at 30°C in LB broth and agar (BD), and phage were propagated by the soft-agar overlay method (3). The morphology of Moabite was determined by samples negatively stained with 2% (wt/vol) uranyl acetate and imaged by transmission electron microscopy at the Texas A&M University Microscopy and Imaging Center (4). The genomic DNA for Moabite was purified with the Promega Wizard DNA clean-up kit according to the modification in the shotgun library preparation protocol given by Summer (5), and then genomic libraries were generated with an Illumina TruSeq nano low-throughput kit. Prepared genomic DNA was sequenced using an Illumina MiSeq platform with 250-bp paired-end reads. We used FastQC (http://www.bioinformatics.babraham.ac.uk/projects/fastqc/) to quality control the 413,089 total reads in the phage-containing index. These reads were trimmed by the FASTX-Toolkit v0.0.14 (http://hannonlab.cshl.edu/fastx_toolkit/). Assembly into a single contig at 287-fold coverage was accomplished with SPAdes v3.5.0, with default parameters (6). The contig was confirmed to be complete by PCR (forward, 5′-CCTGCGTATGTATTCCTGGATAA-3′; reverse, 5′-TTCTTGGTGACATCGTGGTC-3′ primers) and Sanger sequencing. Gene prediction was achieved using GLIMMER v3.0 and MetaGeneAnnotator v1.0 (7, 8). tRNA genes were found with ARAGORN v2.36 (9). The presence of rho-independent terminators was predicted with TransTermHP v2.09 (10). Gene functions were predicted using InterProScan v5.22-61, TMHMM v2.0, and BLAST v2.2.31, with a minimum expectation cutoff of 0.001 against the NCBI nonredundant, UniProtKB Swiss-Prot, and TrEMBL databases (11–14). HHpred with ummiclust30_2018_08 for multiple-sequence alignment (MSA) generation and PDB_mmCIF70 for modeling in the HHsuite v3.0 release provided supplementary evidence for functional prediction (15). Whole-genome sequence identities were calculated with progressiveMauve v.2.4.0 (16). These annotation tools are available on the Center for Phage Technology Galaxy and Web Apollo instances (https://cpt.tamu.edu/galaxy-pub) (17, 18).

Moabite is a myophage with a 273,933-bp genome, 340 predicted protein-coding genes, a G+C content of 46.8%, and a coding density of 94.1%. Functions were predicted for 111 coding regions. The G+C content on Moabite is lower than that of its host, S. marcescens, which has G+C contents ranging from 50.9% to 59.6%, depending on the strain (19). PhageTerm predicts that Moabite uses a headful packaging mechanism, and the genome was reopened in front of the terminase genes (20). From the BLASTp analysis, Moabite shares 312 proteins with *Serratia* phage 2050HW (GenBank accession no. MF285618), and progressiveMauve shows overall 93.57% nucleotide identity with the same phage (21). Unlike for 2050HW, the i-spanin/o-spanin (NCBI accession no. QDB71172 and QDB71173, respectively) and endolysin (NCBI accession no. QDB71048) genes were predicted for Moabite, but no holin gene was positively identified based on sequence similarity.

### Data availability.

The genome sequence and associated data for phage Moabite were deposited under GenBank accession no. MK994515, BioProject accession no. PRJNA222858, SRA accession no. SRR8869230, and BioSample accession no. SAMN11360396.
